# Sex differences in the factors that affect medical lethality in elderly suicide attempters

**DOI:** 10.3389/fpsyt.2023.1260295

**Published:** 2023-11-29

**Authors:** HeungKyu Kim, Seongho Min, Joung-Sook Ahn, Hyun Kim, Yong Sung Cha, Jinhee Lee, Min-Hyuk Kim

**Affiliations:** ^1^Department of Psychiatry, Seoul Metropolitan Eunpyeong Hospital, Seoul, Republic of Korea; ^2^Wonju Mental Health Center, Wonju, Republic of Korea; ^3^Department of Psychiatry, Yonsei University Wonju College of Medicine, Wonju, Republic of Korea; ^4^Department of Emergency Medicine, Yonsei University Wonju College of Medicine, Wonju, Republic of Korea

**Keywords:** sex difference, old age, suicide attempt, lethality, suicide intent

## Abstract

**Objectives:**

This study aimed to identify sex differences in the factors that affect medical lethality in elderly suicide attempters.

**Methods:**

A total of 253 elderly suicide attempters and 351 middle-aged attempters (comparison group) who visited the emergency room at a general hospital were included. The sociodemographic and clinical characteristics of the patients were investigated. The Chi-squared test and logistic regression analysis were performed. And Spearman’s correlation coefficient was calculated.

**Results:**

In older males, the risk of high lethality was lower when attempting suicide due to the loss of family members [adjusted odds ratio (AOR): 0.08]. The risk increased as the intent to die became more certain (some AOR: 11.31, certain AOR: 28.75), and this association became more pronounced with age (rho middle-aged: 0.329; young-old: 0.387; old-old: 0.415). In older females, the risk was lower when employed (AOR: 0.28). The method of suicide attempt also affected lethality (agricultural chemicals AOR: 3.71; psychiatric medication AOR: 0.31).

**Conclusion:**

Sex differences in the factors that affect medical lethality were identified among elderly suicide attempters. In particular, medical lethality can be predicted by the degree of suicide intention in older males. These findings will help to establish more efficient preventive strategies with specific targets.

## 1 Introduction

Suicide is a global health problem, with around 700,000 people dying from suicide every year (1). In Korea, the suicide rate (24.6/100,000) in 2019 was more than twice the average of that in Organization for Economic Co-operation and Development member countries (11/100,000). In particular, the suicide rate among the elderly (65+ years, average: 17.2/100,000, Korea: 46.6/100,000) has been reported to be higher than that in the general population. Furthermore, the suicide rate in Korea tends to increase with age (60s: 33.7; 70s: 46.2; 80s: 67.4 per 100,000) (2). This may actually be worse due to the underestimation problem of not counting a death as a suicide in the absence of clear evidence, particularly in the elderly population (3, 4). The reason for the high suicide rate among the elderly is likely to be that they are physically more vulnerable than other ages, plan suicide with a stronger will, and attempt suicide in more dangerous ways in harder-to-find places (5, 6). Suicide attempts, even if they do not lead to death, often result in physical damage and disability and are associated with high medical and socioeconomic costs. Therefore, in countries such as Korea, where the elderly population is rapidly increasing (7) and the elderly suicide rate is particularly high, it is necessary to identify related factors so that interventions can be established.

The lethality of a suicide attempt has been previously defined and studied in terms of the severity of the medical condition, severity predicted by the attempter or medical professional, and type of ward used (8–13). These studies reported that lethality was affected by factors such as the method of the attempt, gender, drinking status, rescue potential, help-seeking, medical infrastructure, depression, intent to die, communication ability, and impulsivity. In particular, intent to die has often been found to be related to or is important as a predictor of lethality (9–11, 14). Nevertheless, some studies have shown no association between intent and lethality of attempt (15, 16) or that the relationship disappears over time (9). These varied results may be due to the different compositions of the research groups, the inability to control mediators, or different definitions of terms such as intent and lethality. The degree of physical damage caused by a suicide attempt not only affects treatment duration, cost, and intensity but also has clinical significance in predicting subsequent reattempts or death (12, 13, 17). Understanding the relationship between intent and lethality can provide a basis for determining the type and intensity of interventions when a patient’s suicidal intent is identified.

The characteristics of suicide attempts, including lethality, vary according to sex and age. Medical lethality is associated with rescue potential and suicidal intent in females but not associated with intent in males (8). However, in a study with an elderly population, the relationship between lethality and suicidal intent was significant only in males (14). A suicide prevention program for depressed elderly people in Japan was effective in females but not males (18). Therefore, it is important to distinguish subgroups when studying the lethality caused by suicide attempts. This would help in the establishment of specific prevention strategies that work effectively in each subgroup, enable the appropriate allocation of medical resources, and the creation of efficient suicide prevention policies. However, there is still insufficient research on lethality that distinguishes the subgroups. This study aimed to identify sex differences in the factors that affect medical lethality in elderly suicide attempters and to verify whether the relationship between intent to die and lethality varies according to sex.

## 2 Methods

### 2.1 Participants

This study was conducted from 2009 to 2015 on elderly (60+ years) and middle-aged suicide attempters (45–59 years) who visited the emergency room of a hospital in Wonju City, South Korea (19). This is the only tertiary general hospital in the area with sufficient diagnostic and therapeutic resources for injuries caused by suicide attempts, to which most suicide attempters requiring medical intervention are transferred. Only the first suicide attempt was included in the study for cases that reattempted suicide during the study period.

### 2.2 Data collection and measurement

Psychiatric residents and research assistants (nurses, social workers) familiar with the research objectives and evaluation methods collected information from the suicide attempters and their caregivers after arriving at the emergency room and filled in a Crisis Management and Evaluation Record. This document included sociodemographic characteristics such as age, sex, education, marital status, employment, income, and religion; clinical characteristics such as acute/chronic illness and disability, diagnosis according to the DSM-IV-TR (Diagnostic and Statistical Manual of Mental Disorders, Fourth Edition, Text Revision), current psychiatric treatment, and psychiatric history; suicide attempt-related characteristics such as suicide attempt history, family history of suicide, drinking status, motivation, method, intent to die, suicide note, medical lethality and help-seeking. As there are no commonly accepted criteria for defining acute and chronic illness and disability, these were initially classified according to clinical judgment, and the suitability of this classification was reviewed at a case conference. “Intent to die” was classified into three levels (little, some, certain) based on the attempter’s report, and the caregiver’s report was also considered. Medical lethality was classified into three levels (mild, moderate, and severe) based on the clinical judgment of an emergency medicine physician, considering the physical damage and the intensity of medical treatment required.

In situations where the suicide attempter was unable to provide information (e.g., unconsciousness or due to medical treatment), information was obtained from a caregiver, and the suicide attempter was interviewed later. In cases where the reports from the informants did not match, the attempters’ reports were given priority. A weekly case conference was held during the study period to review all records and improve measurement accuracy and interrater reliability. The conference involved two psychiatric specialists, residents, and research assistants who conducted the interviews. Inaccurate or insufficient records were revised through additional interviews and a review of the medical records. This study was approved by the Research Ethics Committee of Yonsei University Wonju College of Medicine, and the requirement for written consent was waived (YMRS-15-09-097).

### 2.3 Statistical analyses

A Chi-square test was performed to identify the variables associated with medical lethality due to suicide attempts. For statistically significant variables in the elderly group, a logistic regression analysis was performed and adjusted for education and monthly income. When medical lethality was classified into three categories, the severe group was relatively small, and there were many cases in which the value of a specific variable became zero, making regression analysis impossible. Therefore, the moderate and severe groups were combined into a mod/sev group and compared with the mild group. “Intent to die” and “medical lethality,” which showed prominent association, were analyzed using Spearman’s correlation coefficient. The age of the elderly attempters was grouped (Young-old: 60–74 years; Old-old: 75+). A *p* < 0.05 was considered statistically significant. The statistical analysis was performed using IBM SPSS Statistics (version 23.0; Armonk, NY, United States).

## 3 Results

A total of 604 suicide attempters (288 males and 316 females) were included in the analysis, of whom 253 were elderly (139 males and 114 females) and 351 were middle-aged (149 males and 202 females) (Table 1). Males ranged in age from 45 to 92 years with an average of 61.7 ± 11.9 (elderly: 60–92, 72.4 ± 7.7; middle-aged: 45–59, 51.8 ± 4.0), and females ranged in age from 45 to 98 years with an average of 59.1 ± 12.1 (elderly: 60–98, 73.1 ± 8.6; middle-aged: 45–59, 51.2 ± 3.9). The mild, moderate, and severe groups (medical lethality) included 39 (28.1%), 64 (46.0%), and 36 (25.9%) older males, and 37 (32.5%), 62 (54.4%), and 15 (13.2%) older females, respectively. In middle-aged males, it was 55 (36.9%), 77 (51.7%), and 17 (11.4%), and in middle-aged females, it was 95 (47.0%), 91 (45.0%), and 16 (7.9%), respectively. The proportion of high lethality (mod/sev) was higher in the elderly group than in the middle-aged group for both sexes (middle-aged males: 63.1%; older males: 71.9%; middle-aged females: 53%; and older females: 67.5%).

**Table 1:** Age distribution and medical lethality of subgroups.

Sex	Males (*N* = 288)		Females (*N* = 316)	
Age group	Middle age (*N* = 149)	Old age (*N* = 139)	Middle age (*N* = 202)	Old age (*N* = 114)
Age, years				
Range	45–59	60–92	45–59	60–98
Mean ± SD	51.8 ± 4.0	72.4 ± 7.7	51.2 ± 3.9	73.1 ± 8.6
Medical lethality, n (%)				
Mild	55 (36.9%)	39 (28.1%)	95 (47.0%)	37(32.5%)
Mod/Sev†	94 (63.1%)	100 (71.9%)	107 (53%)	77 (67.5%)
Moderate	77 (51.7%)	64 (46%)	91 (45.0%)	62 (54.4%)
Severe	17 (11.4%)	36 (25.9%)	16 (7.9%)	15 (13.2%)

†The moderate and severe groups were combined into a mod/sev group.

Tables 2, 3 show the sociodemographic, clinical, and suicide attempt-related characteristics and their association with the dependent variable, medical lethality. In older females, lethality was associated with employment status (χ^2^ = 4.71, *p* = 0.03), with 24.3% of the mild lethality group being unemployed compared to 45.5% of the mod/sev group. In older males, lethality was lower when the motivation for the suicide attempt was bereavement (χ^2^ = 4.47; *p* = 0.04). Most older adults (>80%) attempted suicide by poisoning, with more use of psychiatric medication (χ^2^ = 4.82, *p* = 0.03) in the mild group and more use of pesticides and herbicides (χ^2^ = 5.12, *p* = 0.02) in the mod/sev group in older females. However, these substances were not associated with lethality in older males, and pesticide and herbicide use were the most common in both lethality groups (>50%). The association between intent to die and lethality was significant in middle-aged males (χ^2^ = 14.95, *p* < 0.01), middle-aged females (χ^2^ = 22.55, *p* < 0.01), and older males (χ^2^ = 28.99, p < 0.01), but not in older females (χ^2^ = 4.42, *p* = 0.11).

**Table 2:** Sociodemographic and clinical characteristics of the males.

Age and sex	Middle-aged males (*n* = 149)						Older males (*n* = 139)					
Medical lethality	Mild (*n* = 55)						Mod/Sev (*n* = 94)			Mild (*n* = 39)						Mod/Sev (*n* = 100)		
	*n*	%	*n*	%	χ^2^	*p*	*n*	%	*n*	%	χ^2^	*p*
Education												
≤Elementary school	14	25.9	22	25.0	0.02	0.99	29	74.4	70	72.2	0.31†	0.94
Middle/High school	35	64.8	58	65.9			9	23.1	22	22.7		
≥College	5	9.3	8	9.1			1	2.6	5	5.2		
Monthly income, US$												
≤1,000	22	40.7	38	46.9	**9.98**	**0.02**	26	78.8	68	73.9	1.52†	0.73
1,000–2000	21	38.9	13	16.0			6	18.2	14	15.2		
2,000–3,000	8	14.8	21	25.9			1	3.0	6	6.5		
≥3,000	3	5.6	9	11.1			0	0	4	4.3		
Marital status												
Single/never married	6	10.9	7	7.7	3.21	0.20	0	0	1	1	0.87†	0.75
Married/cohabitation	30	54.5	63	69.2			32	82.1	75	75.8		
Separated/divorced/widowed	19	34.5	21	23.1			7	17.9	23	23.2		
Job												
No	14	25.5	30	32.6	0.84	0.36	21	53.8	54	54.0	<0.01	0.99
Yes	41	74.5	62	67.4			18	46.2	46	46.0		
Religion												
No	32	59.3	56	65.9	0.62	0.43	24	63.2	62	66.0	0.09	0.76
Yes	22	40.7	29	34.1			14	36.8	32	34.0		
Physical illness												
None	40	74.1	56	60.2	3.31†	0.36	15	38.5	25	25.5	4.26	0.24
Acute illness	2	3.7	3	3.2			3	7.7	4	4.1		
Chronic illness without disability	4	7.4	13	14.0			7	17.9	31	31.6		
Chronic illness with disability	8	14.8	21	22.6			14	35.9	38	38.8		
Smoking												
Never	9	18.4	16	22.9	0.43	0.81	15	40.5	32	41.6	1.71	0.43
Ex-smoker	6	12.2	7	10.0			13	35.1	19	24.7		
Current smoker	34	69.4	47	67.1			9	24.3	26	33.8		
Current psychiatric treatment												
No	37	74.0	72	82.8	1.50	0.22	30	81.1	80	87.9	1.02	0.31
Yes	13	26.0	15	17.2			7	18.9	11	12.1		
Diagnosis, DSM-IV-TR§												
Anxiety disorder	1	2.4	1	1.3	0.23	0.63	0	0	3	3.8	1.19	0.28
Depressive disorder	14	34.1	33	41.8	0.66	0.42	17	56.7	45	57.7	0.01	0.92
Bipolar disorder	0	0	0	0	N/A	N/A	0	0	1	1.3	0.39	0.53
Schizophrenia	2	4.9	2	2.5	0.46	0.50	0	0	0	0	N/A	N/A
Substance use disorder	17	41.5	29	36.7	0.26	0.61	1	3.3	12	15.4	2.97	0.09
Dementia	0	0	0	0	N/A	N/A	4	13.3	5	6.4	1.36	0.24
Adjustment disorder	6	14.6	17	21.5	0.83	0.36	9	30.0	13	16.7	2.38	0.12
Others	1	2.4	2	2.5	<0.01	0.98	0	0	0	0	N/A	N/A
Family psychiatric history												
No	47	87.0	79	89.8	0.25	0.62	34	91.9	85	90.4	0.07	0.79
Yes	7	13.0	9	10.2			3	8.1	9	9.6		
Alcohol consumption before attempt												
No	16	29.1	30	33.7	0.33	0.56	21	53.8	51	52.0	0.04	0.85
Yes	39	70.9	59	66.3			18	46.2	47	48.0		
Motivation for suicide attempt§												
Interpersonal problem	33	60.0	44	48.4	1.87	0.17	17	44.7	34	35.8	0.92	0.34
Job-related problem	10	18.2	22	24.2	0.72	0.40	2	5.3	9	9.5	0.63	0.43
Economic problem	18	32.7	40	44.0	1.81	0.18	3	7.9	19	20.0	2.88	0.09
Illness-related problem	5	9.1	11	12.1	0.32	0.57	17	44.7	35	36.8	0.71	0.39
Loss of a family member	4	7.3	3	3.3	1.19	0.28	4	10.5	2	2.1	**4.47**	**0.04**
Abuse	0	0	1	1.1	0.61	0.44	0	0	1	1.1	0.40	0.53
Legal problem	0	0	1	1.1	0.61	0.44	2	5.3	0	0	5.08	0.02
Method of suicide attempt												
Poisoning§	41	74.5	64	68.1	0.70	0.40	33	84.6	84	84.0	0.01	0.93
Analgesics	1	1.8	1	1.1	0.15	0.70	2	5.1	0	0	**5.20**	**0.02**
Psychiatric medications	12	21.8	18	19.1	0.15	0.70	7	17.9	21	21.0	0.16	0.69
Agricultural chemicals	22	40.0	40	42.6	0.09	0.76	23	59.0	58	58.0	0.01	0.92
Others	11	20.0	19	20.2	<0.01	0.98	1	2.6	8	8.0	1.37	0.24
Charcoal burning	6	10.9	15	16.0	0.73	0.39	0	0	6	6.0	2.45	0.12
Hanging	5	9.1	8	8.5	0.02	0.90	2	5.1	6	6.0	0.04	0.84
Jumping from high place/Drowning	0	0	1	1.1	0.59	0.44	0	0	0	0	N/A	N/A
Injury by sharp object	2	3.6	6	6.4	0.52	0.47	3	7.7	2	2.0	2.62	0.11
Others	1	1.8	0	0	1.72	0.19	1	2.6	2	2.0	0.04	0.84
Suicide note												
No	48	87.3	75	82.4	0.61	0.44	38	100.0	90	93.8	2.49	0.12
Yes	7	12.7	16	17.6			0	0	6	6.3		
Intent to die												
Little	13	23.6	9	9.6	**14.95**	**<0.01**	12	30.8	3	3.0	**28.9**	**<0.01**
Some	24	43.6	24	25.5			12	30.8	18	18.0		
Certain	18	32.7	61	64.9			15	38.5	79	79.0		
Seek help												
No	30	56.6	62	71.3	4.78	0.09	25	65.8	73	73.0	3.36	0.19
Clues provided	7	13.2	12	13.8			1	2.6	8	8.0		
Yes	16	30.2	14	14.9			12	31.6	19	19.0		

†Fisher’s exact test. §Multiple selection allowed. N/A, not available; mod/sev, moderate and severe. The n value of each variable is different due to the missing data. Bold values mean that they are statistically significant (*p* < 0.05).

**Table 3:** Sociodemographic and clinical characteristics of the females.

Age and sex	Middle-aged females (*n* = 202)						Older females (*n* = 114)					
Medical lethality	Mild (*n* = 95)						Mod/Sev (*n* = 107)			Mild (*n* = 37)						Mod/Sev (*n* = 77)		
	*n*	%	*n*	%	χ^2^	*p*	*n*	%	*n*	%	χ^2^	*p*
Education												
≤Elementary school	26	28.0	34	34.0	1.11	0.58	29	80.6	62	81.6	0.82†	0.81
Middle/high school	59	63.4	60	60.0			7	19.4	12	15.8		
≥College	8	8.6	6	6.0			0	0	2	2.6		
Monthly income, US$												
≤1,000	27	32.1	32	35.6	2.28	0.52	19	52.8	32	48.5	0.33†	0.98
1,000–2000	26	31.0	28	31.1			10	27.8	20	30.3		
2,000–3,000	19	22.6	13	14.4			3	8.3	7	10.6		
≥3,000	12	14.3	17	18.9			4	11.1	7	10.6		
Marital status												
Single/never married	2	2.1	2	1.9	1.10†	0.63	0	0	3	3.9	5.91†	0.05
Married/cohabitation	68	72.3	83	78.3			24	64.9	31	40.8		
Separated/divorced/widowed	24	25.5	21	19.8			13	35.1	42	55.3		
Job												
No	11	11.6	22	20.6	2.97	0.09	9	24.3	35	45.5	**4.71**	**0.03**
Yes	84	88.4	85	79.4			28	75.7	42	54.5		
Religion												
No	45	47.4	54	52.4	0.51	0.48	16	43.2	30	39.5	0.15	0.70
Yes	50	52.6	49	47.6			21	56.8	46	60.5		
Physical illness												
None	56	60.2	71	67	**9.54**	**0.02**	12	32.4	17	22.7	1.89	0.60
Acute illness	8	8.6	1	0.9			2	5.4	6	8.0		
Chronic illness without disability	22	23.7	19	17.9			11	29.7	20	26.7		
Chronic illness with disability	7	7.5	15	14.2			12	32.4	32	42.7		
Smoking												
Never	54	69.2	64	68.1	0.03	0.87	26	83.9	55	88.7	0.87†	0.76
Ex-smoker	0	0	0	0			1	3.2	2	3.2		
Current smoker	24	30.8	30	31.9			4	12.9	5	8.1		
Current psychiatric treatment												
No	65	72.2	68	69.4	0.18	0.67	23	67.6	54	76.1	0.83	0.36
Yes	25	27.8	30	30.6			11	32.4	17	23.9		
Diagnosis, DSM-IV-TR§												
Anxiety disorder	0	0	0	0	N/A	N/A	0	0	0	0	N/A	N/A
Depressive disorder	50	65.8	50	58.1	0.99	0.32	22	73.3	47	82.5	0.99	0.32
Bipolar disorder	2	2.6	3	3.5	0.09	0.75	1	3.3	1	1.8	0.22	0.64
Schizophrenia	0	0	3	3.5	2.70	0.1	1	3.3	1	1.8	0.22	0.64
Substance use disorder	6	7.9	11	12.8	1.03	0.31	1	3.3	2	3.5	<0.01	0.97
Dementia	0	0	0	0	N/A	N/A	0	0	0	0	N/A	N/A
Adjustment disorder	18	23.7	19	22.1	0.06	0.81	7	23.3	7	12.3	1.78	0.18
Others	1	1.3	1	1.2	<0.01	0.93	0	0	0	0	N/A	N/A
Family psychiatric history												
No	85	91.4	87	87.0	0.96	0.33	36	97.3	68	91.9	1.22	0.27
Yes	8	8.6	13	13.0			1	2.7	6	8.1		
Alcohol consumption before attempt												
No	33	34.7	42	39.6	0.51	0.48	28	75.7	64	84.2	1.19	0.27
Yes	62	65.3	64	60.4			9	24.3	12	15.8		
Motivation for suicide attempt§												
Interpersonal problem	63	67.7	69	67.0	0.01	0.91	19	51.4	26	35.1	2.69	0.10
Job-related problem	6	6.5	13	12.6	2.13	0.15	0	0	2	2.7	1.02	0.31
Economic problem	13	14.0	20	19.4	1.03	0.31	3	8.1	6	8.1	<0.01	>0.99
Illness-related problem	5	5.4	6	5.8	0.02	0.89	15	40.5	34	45.9	0.29	0.59
Loss of a family member	2	2.2	2	1.9	0.01	0.92	3	8.1	8	10.8	0.20	0.65
Abuse	0	0	1	1.0	0.91	0.34	0	0	0	0	N/A	N/A
Legal problem	0	0	1	1.0	0.91	0.34	0	0	0	0	N/A	N/A
Method of suicide attempt												
Poisoning§	73	76.8	81	75.7	0.04	0.85	31	83.8	69	89.6	0.79	0.38
Analgesics	3	3.2	4	3.7	0.05	0.82	0	0	1	1.3	0.49	0.49
Psychiatric medications	41	43.2	30	28.0	**5.05**	**0.03**	21	56.8	27	35.1	**4.82**	**0.03**
Agricultural chemicals	20	21.1	34	31.8	2.95	0.09	7	18.9	31	40.3	**5.12**	**0.02**
Others	9	9.5	17	15.9	1.85	0.17	5	13.5	11	14.3	0.01	0.91
Charcoal burning	7	7.4	9	8.4	0.08	0.78	4	10.8	2	2.6	3.38	0.07
Hanging	5	5.3	3	2.8	0.8	0.37	1	2.7	3	3.9	0.11	0.75
Jumping from high place/Drowning	1	1.1	4	3.7	1.50	0.22	0	0	0	0	N/A	N/A
Injury by sharp object	8	8.4	9	8.4	<0.01	0.99	1	2.7	2	2.6	0.01	0.97
Others	1	1.1	1	0.9	0.01	0.93	0	0	1	1.3	0.49	0.49
Suicide note												
No	88	94.6	100	94.3	0.01	0.93	35	94.6	70	95.9	0.09	0.76
Yes	5	5.4	6	5.7			2	5.4	3	4.1		
Intent to die												
Little	38	40.0	25	23.4	**22.55**	**<0.01**	9	24.3	8	10.4	4.42	0.11
Some	38	40.0	26	24.3			10	27.0	19	24.7		
Certain	19	20.0	56	52.3			18	48.6	50	64.9		
Seek help												
No	56	62.2	65	62.5	0.01	0.99	24	68.6	63	82.9	4.36†	0.09
Clues provided	11	12.2	13	12.5			4	11.4	2	2.6		
Yes	23	25.6	26	25.0			7	20.0	11	14.5		

†Fisher’s exact test. §Multiple selection allowed. N/A, not available; Mod/Sev, moderate and severe. The n value of each variable is different due to the missing data. Bold values mean that they are statistically significant (*p* < 0.05).

Table 4 presents the odds ratios (OR) and confidence intervals (CI) for higher lethality in elderly individuals. In elderly males, the risk of higher lethality was lower when the motivation for the suicide attempt was the loss of a family member (OR: 0.18, CI: 0.03–1.04; Adjusted OR (AOR): 0.08, CI: 0.01–0.74), while increased risk was observed when the intent was “some” (OR: 6.00, CI: 1.39–25.86; AOR: 11.31, CI: 1.88–68.25) or “certain” (OR: 21.07, CI: 5.31–83.77; AOR: 28.75, CI: 5.17–160.78). In older females, the risk of higher lethality was lower when employed (OR: 0.39, CI: 0.16–0.93; AOR: 0.28, CI: 0.11–0.75) and when attempting suicide with psychiatric medication (OR: 0.41, CI: 0.19–0.92; AOR: 0.31, CI: 0.12–0.78), whereas the risk was higher when attempting suicide with pesticides or herbicides (OR: 2.89, CI: 1.13–7.40; AOR: 3.71, CI: 1.31–10.49). In elderly females, an increased risk was observed when the intent was “certain”; however, this was not significant after adjusting for education and monthly income (OR: 3.13, CI: 1.05–9.33; AOR: 2.68, CI: 0.84–8.54).

**Table 4:** Factors that affect medical lethality.

	Middle-aged males		Middle-aged females		Older males		Older females	
	UOR (CI)	AOR (CI)	UOR (CI)	AOR (CI)	UOR (CI)	AOR (CI)	UOR (CI)	AOR (CI)
Job	0.71 (0.33–1.49)	0.50 (0.20–1.23)	0.51 (0.23–1.11)	0.47 (0.19–1.17)	0.99 (0.47–2.09)	0.98 (0.42–2.28)	0.39* **(0.16–0.93)**	0.28* **(0.11–0.75)**
Loss of a family member†	0.44 (0.09–2.02)	0.16 (0.02–1.67)	0.90 (0.12–6.53)	1.76 (0.19–15.88)	0.18 (0.03–1.04)	0.08* **(0.01–0.74)**	1.37 (0.34–5.52)	1.20 (0.27–5.32)
Analgesics§	0.58 (0.04–9.47)	N/A	1.19 (0.26–5.46)	1.40 (0.29–6.82)	N/A	N/A	N/A	N/A
Psychiatric medication§	0.85 (0.37–1.93)	1.11 (0.44–2.77)	0.51* **(0.29–0.92)**	0.59 (0.31–1.12)	1.22 (0.47–3.14)	1.43 (0.47–4.36)	0.41* **(0.19–0.92)**	0.31* **(0.12–0.78)**
Agricultural chemicals§	1.11 (0.57–2.19)	1.01 (0.47–2.16)	1.75 (0.92–3.31)	1.51 (0.72–3.17)	0.96 (0.45–2.04)	1.21 (0.52–2.82)	2.89* **(1.13–7.40)**	3.71* **(1.31–10.49)**
Intent to die								
Some‡	1.44 (0.52–4.01)	2.66 (0.80–8.79)	1.04 (0.51–2.12)	1.22 (0.55–2.67)	6.00* **(1.39–25.86)**	11.31** **(1.88–68.25)**	2.14 (0.63–7.26)	2.16 (0.60–7.77)
Certain‡	4.90** **(1.80–13.30)**	6.68** **(2.08–21.45)**	4.48** **(2.17–9.25)**	5.16** **(2.27–11.70)**	21.07** **(5.31–83.77)**	28.75** **(5.17–160.78)**	3.13* **(1.05–9.33)**	2.68 (0.84–8.54)

UOR, unadjusted odds ratio; AOR, adjusted odds ratio, adjusted for education and monthly income; CI, confidence interval; N/A, not available. †Reference: other motivations. §Reference: other methods. ‡Reference: little intent to die. *p < 0.05; **p < 0.01. Bold values mean that they are statistically significant (*p* < 0.05).

**Figure 1:**
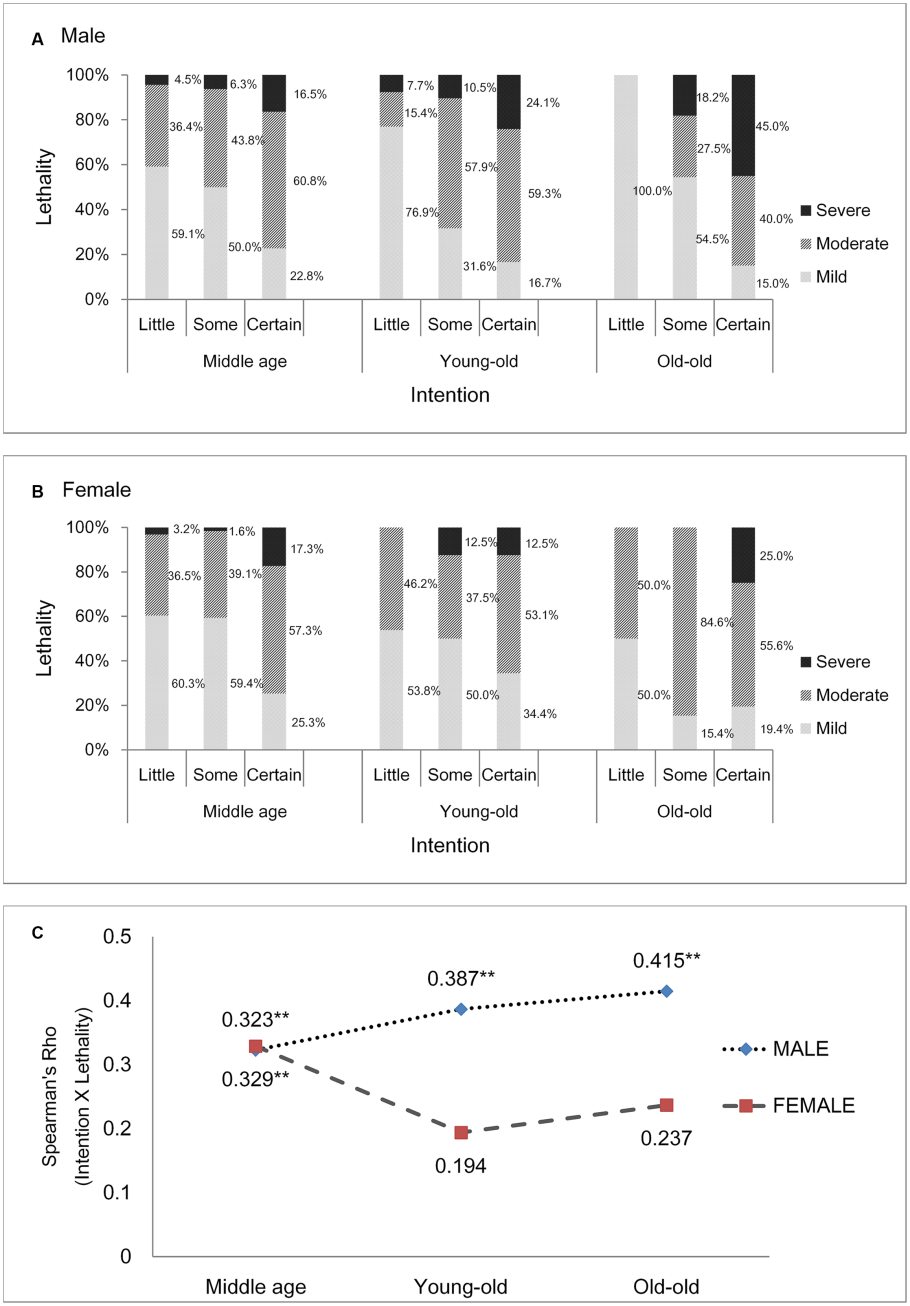
Correlation between intent to die and lethality. **(A)** Males, **(B)** females (bar: proportion of each lethality), **(C)** Spearman’s correlation coefficient. The symbol ** means it is statictically significant (*p* < 0.01).

Figure 1 shows that the relationship between “intent to die” and “medical lethality” changes with age and that there are sex differences in these changes. In males, as intent increases (i.e., little, some, certain), the proportion of mild lethality decreases, and the proportion of severe lethality increases. Furthermore, this phenomenon becomes more pronounced in older age groups. In females, no such trend was observed with increasing age. Figure 1C shows the correlation between “intent to die” and “medical lethality” through Spearman’s correlation coefficient (rho). In males, the correlation between the two variables was significant at all ages, and the correlation coefficient increased as age increased (rho: middle age: 0.323, young old: 0.387, old-old: 0.415). However, in females, a significant correlation (rho: 0.329) was shown only in middle age.

## 4 Discussion

This study aimed to identify the factors that affect medical lethality in elderly suicide attempters and to verify whether the relationship between “intent to die” and “medical lethality” differs by sex. We found that in older males, the risk of lethality was lower when attempting suicide due to the loss of a family member compared to other motivations. The risk increased as intent became more definite, and this association became more pronounced with age. In older females, the risk was lower when employed or when attempting suicide with psychiatric medication and higher when using agricultural chemicals. Physical condition or help-seeking, which were expected to be closely related to lethality, were not significant in either sex.

The association between the intent to die and lethality was significant and increased with age in males. This association was similar in middle-aged females but was no longer present in older females. Factors such as the attempt method, ability to execute the attempt accurately, avoiding detection, time taken for transport, and physical health affect the course from intention to physical damage. However, sufficient research has not yet been conducted on how these factors differ by sex. Dombrovski et al. suggested that hormonal changes from aging affect the frontal cortex and executive function differently by sex, causing older males to be better at acting out their suicidal intent, leading to more significant physical damage (14). They also reported that prefrontal functions, such as reward delay and planning, are associated with lethality (20). It can also be speculated that physical vulnerability in older males allows for damage proportional to the intent to die. The shorter life expectancy of older males indirectly supports their higher vulnerability (21, 22). However, this does not explain why the association between intent and lethality observed in middle-aged females disappears in physically weaker, older females. This finding suggests that suicidal intent should be actively explored in older males, particularly in the older age groups. When a high level of intent is identified, treatment for psychopathology and preventive interventions for suicide should be initiated immediately. However, in older females, even if they are reported to have low suicidal intentions, caution is needed because the damage cannot be expected to be small.

In our study, both male and female elderly attempters most frequently used poison, especially psychiatric medications and agricultural chemicals (pesticides and herbicides), to attempt suicide. Self-poisoning was also the most common method used by elderly attempters in other countries (23, 24). The most frequently used substance is a psychiatric medication that is relatively less harmful than other substances. However, the use of agricultural chemicals is generally low except in a few countries, such as Korea and China (25, 26). In Korea, suicide attempts using agricultural chemicals are more common than in Taiwan and Japan, which have similar economic and sociocultural backgrounds (27, 28), because of limitations in the enactment and implementation of laws that restrict access to such substances (29). In addition, because the elderly population engaged in agriculture in the study area was considerable, it was not difficult for elderly attempters to obtain information about the chemicals and to access and acquire them. This implies that Korea needs to overhaul its laws restricting access and strengthening assessment of suicidality when older people buy agricultural chemicals. It remains unclear why this method, which can directly affect the degree of damage, was not associated with lethality in elderly males. Other aspects, such as the dose of the ingested substance or the intensity of the implementation, should be investigated in future studies. The importance of ascertaining the suicidal intention of older males is further emphasized in that the main determinant of lethality is suicidal intention rather than the method of the attempt.

Unemployment and job loss generally increase male suicide risk; however, the association increases in females with longer follow-up periods and older age (30–32). Similarly, in our study, lethality was low among older employed females. Deterioration in employment status may indirectly affect physical impairment in older adults through reduced self-esteem, depression, and hopelessness. Conversely, having a job may reflect that they are in good health condition. However, there is still no plausible explanation as to why this phenomenon occurs only in females. It is hoped that follow-up studies will shed light on how occupation has a sex-specific effect on these mediators and, eventually, physical damage.

A few studies reported that the impact of spousal death on suicidal behavior is greater in older males, while older females tend to become more affected over time (33, 34). However, this difference could not be confirmed in this study because bereavement, separation, and divorce were grouped into one category of marital status. Our results showed that the risk of high lethality was lower when the motivation for suicide attempt is the loss of a family member in older males. We did not examine whether the lost family member was the spouse. In addition, there were very few participants whose motivation for suicide was the loss of a family member. These altogether hinder us from explaining the results. Further study is needed to replicate our results.

This study had several limitations. First, objective comparison with other studies was challenging since a standardized scale was not used, and the interrater reliability may be low. Changes in cognitive function which are important in geriatric study were not evaluated using an objective scale such as the Mini-Mental Status Examination (MMSE). However, due to time limitations in the emergency department, the use of time-consuming assessment tools for suicide research could not be performed. Second, suicidal intent or related features may have been overreported which would have received less attention prior to the suicide attempt. Cohort studies can compensate for this problem; however, they are expensive due to the low suicide attempt rates within a general cohort. Third, it is difficult to generalize the study results to all older people for several reasons. First, this study had a relatively small sample size and was conducted at a single center. Second, both extremes of the lethality continuum, death and non-transfer, were excluded from the study. Nevertheless, this study was conducted over a long period at a hospital where most suicide attempters requiring medical intervention are transferred within a wide administrative area.

## 5 Conclusion

The suicidal intent reported by older males is closely related to lethality; therefore, appropriate measures are required that increase in importance with age. However, the lethality of suicide attempts in older females cannot be expected to be low, even if their intent is reported to be low; therefore, caution is needed. Compared with other suicidal motivations, bereavement of a family member has a lower risk of lethality in older males. In older females, the risk is lower when employed or attempting suicide with psychiatric medication and higher when attempting suicide with agricultural chemicals. Due to the lack of related studies, providing a sufficient evidence-based explanation for the influencing factors and sex-specific effects is challenging. Further studies with larger sample sizes and cases at both ends of the lethality continuum are needed to enable more detailed analysis.

## Data availability statement

The datasets presented in this article are not readily available because the datasets for this article are not publicly available due to concerns regarding participant/patient anonymity. Requests to access the datasets should be directed to the corresponding author. Requests to access the datasets should be directed to M-HK, mhkim09@yonsei.ac.kr.

## Ethics statement

The studies involving humans were approved by the Research Ethics Committee of Yonsei University Wonju College of Medicine. The studies were conducted in accordance with the local legislation and institutional requirements. Written informed consent for participation was not required from the participants or the participants’ legal guardians/next of kin in accordance with the national legislation and institutional requirements.

## Author contributions

HKK: Investigation, Writing – review & editing, Formal analysis, Writing – original draft. SM: Writing – review & editing, Conceptualization, Project administration, Supervision. J-SA: Writing – review & editing, Investigation. HK: Investigation, Writing – review & editing, Data curation, Methodology. YC: Data curation, Investigation, Methodology, Writing – review & editing. JL: Data curation, Investigation, Methodology, Writing – review & editing, Resources, Validation. M-HK: Investigation, Conceptualization, Supervision, Writing – review & editing.
